# Alerts and Collections for Automating Patients’ Sensemaking and Organizing of Their Electronic Health Record Data for Reflection, Planning, and Clinical Visits: Qualitative Research-Through-Design Study

**DOI:** 10.2196/41552

**Published:** 2023-08-21

**Authors:** Drashko Nakikj, David Kreda, Nils Gehlenborg

**Affiliations:** 1 Department of Biomedical Informatics, Harvard Medical School, Harvard University Boston, MA United States

**Keywords:** patients, electronic health records, sensemaking, pattern detection, data organization, alerts, reports, collections

## Abstract

**Background:**

Electronic health record (EHR) data from multiple providers often exhibit important but convoluted and complex patterns that patients find hard and time-consuming to identify and interpret. However, existing patient-facing applications lack the capability to incorporate automatic pattern detection robustly and toward supporting making sense of the patient’s EHR data. In addition, there is no means to organize EHR data in an efficient way that suits the patient’s needs and makes them more actionable in real-life settings. These shortcomings often result in a skewed and incomplete picture of the patient’s health status, which may lead to suboptimal decision-making and actions that put the patient at risk.

**Objective:**

Our main goal was to investigate patients’ attitudes, needs, and use scenarios with respect to automatic support for surfacing important patterns in their EHR data and providing means for organizing them that best suit patients’ needs.

**Methods:**

We conducted an inquisitive research-through-design study with 14 participants. Presented in the context of a cutting-edge application with strong emphasis on independent EHR data sensemaking, called Discovery, we used high-level mock-ups for the new features that were supposed to support *automatic identification of important data patterns and offer recommendations*—*Alerts*—and *means for organizing the medical records based on patients’ needs, much like photos in albums—Collections*. The combined audio recording transcripts and in-study notes were analyzed using the reflexive thematic analysis approach.

**Results:**

The *Alerts* and *Collections* can be used for raising awareness, reflection, planning, and especially evidence-based patient-provider communication. Moreover, patients desired carefully designed automatic pattern detection with safe and actionable recommendations, which produced a well-tailored and scoped landscape of alerts for both potential threats and positive progress. Furthermore, patients wanted to contribute their own data (eg, progress notes) and log feelings, daily observations, and measurements to enrich the meaning and enable easier sensemaking of the alerts and collections. On the basis of the findings, we renamed *Alerts* to *Reports* for a more neutral tone and offered design implications for contextualizing the reports more deeply for increased actionability; automatically generating the collections for more expedited and exhaustive organization of the EHR data; enabling patient-generated data input in various formats to support coarser organization, richer pattern detection, and learning from experience; and using the reports and collections for efficient, reliable, and common-ground patient-provider communication.

**Conclusions:**

Patients need to have a flexible and rich way to organize and annotate their EHR data; be introduced to insights from these data—both positive and negative; and share these artifacts with their physicians in clinical visits or via messaging for establishing shared mental models for clear goals, agreed-upon priorities, and feasible actions.

## Introduction

### Background

During the last decade in the United States, efforts have been made to allow patients to access electronic health record (EHR) data from their providers. Although big strides have been successfully made toward setting up standards and policies to do that [1,2], less progress has been made in understanding how to aid patients in making sense of their EHR data and present them in useful and actionable ways.

Patients still predominantly access their EHR data through patient portals, usually tethered to providers’ EHR systems [3]. These tools have made some accomplishments in incorporating features that allow patients to inspect their data [4]; however, these are primarily overview and look-up features that are not designed for deeper engagement in data exploration and finding important, interesting patterns independently [5]. In addition, patient portals enable access to EHR data from a single provider, mostly with poor interoperability capabilities. This results in patients not being able to access their data from multiple providers or health care systems at the same time [6]. Such limitations usually cause patients to manually aggregate their siloed data in a time-consuming and frustrating process that is often left unfinished. Consequently, there is a widespread problem of patients’ EHR data being fragmented across multiple providers and patients having difficulties in making sense of these data in their siloed patient portals. Numerous studies point out the negative consequences for patients of these issues, emphasizing overwhelmingness with the amount of available but fragmented data [6,7], lack of patients’ and their physicians’ awareness of existing EHR data [6,8], underwhelming features that support patients’ independent identification of patterns in their EHR data that reflect or are tied to health problems [3,9], patients’ inability to refer back to the sensemaking activities they conducted in their portals [6], and lack of capabilities for effective evidence-based communication with their physicians [6]. These shortcomings manifest in a plethora of more concrete problems: limitations in knowing the complete picture of the patients’ medical history and ongoing health issues leading to redundant and duplicate tests, medical errors, and suboptimal decision-making and treatment; the lack of self-advocacy and poor patient-provider communication bringing difficulties in explaining the problem, setting goals, providing context and evidence, and devising care plans with referable, clear, and understandable actions; and challenges in transition of care and solving complex problems that require the engagement of multiple specialists across multiple institutions. To overcome these problems, patients have to be able to efficiently aggregate and make sense of their EHR data and reliably communicate the insights to their physicians.

Although improvements in the various patient portal usefulness and usability categories may be possible to address these goals, we decided to take a novel approach for presenting patients with their EHR data. We hope that a new model of interacting with the EHR data could provide a different perspective to the patients and, thus, open the gates for more efficient and significant improvements in supporting the sensemaking of their EHR data. To achieve this, we relied on established sensemaking theories, fundamental sensemaking activities, and principles for the collaborative determination of diagnosis and treatment. Previous research has explored how patients make sense of their personal health data for disease management, anchored in the sensemaking data frame theory [10]. This theory posits that, for an open question, individuals collect relevant data that constitute a frame [11]. Within that frame, they try to find patterns that could contribute to answering the question. During this process, the frames can be updated by adding new data, eliminating existing data, or extending to new frames. Circling back to the previously mentioned work on disease management, researchers focused on diabetes and found that frames were primarily formulated to find cause-effect relationships. These frames were grounded in contextual (eg, exercise) and clinical (eg, insulin dosage) factors for the purpose of describing different ways in which they affect or could affect the outcome measures (eg, blood glucose numbers). However, finding correlations is not the only activity that is important for sensemaking of health data. Previous research particularly has focused on the basic activities patients engage in when they are trying to make sense of their patient-generated data (PGD)—extreme values, trends, and correlations, among others [10,12]. In contrast, other work has explored patients’ sensemaking activities for their EHR data from multiple providers (eg, hospitals and clinics), such as prevalence, frequency, co-occurrence, and pre-post analysis of clinical events [9]. Furthermore, researchers have emphasized the importance of PGD in communication during clinical appointments. The PGD were perceived as facilitators to set boundaries within which parsing the space for diagnosis and treatment will take place by the physician in collaboration with the patient [13,14]. Analogous to this, we could envision EHR data being used to set similar types of boundaries. Within these boundaries, patients and their physicians can engage in new types of sensemaking activities that involve EHR data.

Motivated by this background, we can offer capabilities that allow patients to organize their EHR data in collections (ie, data frames) that can be tailored to answering their specific information needs and support sensemaking regarding their health. These collections can be manually generated by the patients and be subject to independent sensemaking activities for pattern detection. However, EHR data that come from multiple providers exhibit simple patterns, which almost anybody can find and recognize, and convoluted and complex patterns that even the greatest patient experts cannot identify and interpret [8]. Therefore, it is not always clear to patients which questions to ask (ie, what patterns to look for [15]). In addition, it can be difficult to identify such patterns completely manually [16] in a process that could be very time-consuming and requires substantial medical knowledge, analytical skills, and motivation [7,8]. Therefore, a different type of data frame can be automatically generated by the system based on patterns in the EHR data and well-established clinical guidelines. On the basis of this argument, it appears that there is a promising approach toward supporting the sensemaking of EHR data inspired by the data frame theory. However, it has only been partially addressed by contemporary solutions and existing research.

In recent years, efforts have been made to build applications that help patients make sense of their EHR data from multiple providers. Although this idea is still in its inception, interesting new solutions have emerged, such as Apple Health Records [17], iBlueButton [18], and OneRecord [19] for mobile devices and 1upHealth [20] and Discovery [21] for desktop. These solutions lay out medical records by the date they were entered, type (eg, medications and laboratory test results), or provider. On the basis of this predetermined structure, they allow users to independently explore and find patterns of interest in their EHR data, such as increasing and decreasing trends in laboratory test results or vital signs, periodicity of immunizations or medications, and co-occurrence of medical events in the same day or time interval, just to name a few. Similar to patient portals, these applications feature patient-facing alerts that are mostly focused on appointment reminders, above or below normal values in laboratory test results, or medication refill [22]. Furthermore, they are not specifically designed to support ongoing, independent sensemaking of EHR data but rather to prevent immediate issues. Despite these advancements in sensemaking support, contemporary solutions have some noticeable limitations.

Existing solutions do not allow the patient to organize their EHR data based on their information needs in a personalized way—by acute health issues or ongoing problems, for example. However, organizing personal data is a key dimension of information management and highly desired among patients [23-25]. In addition, contemporary applications offer no means of referring back to the sensemaking process at later times. This leaves patients with the tedious and frustrating burden of repeatedly collecting relevant information for frequent and related information needs, repeating the inferences over those data, or recreating mental notes. Furthermore, in cases where automatic support for surfacing trends and patterns in the EHR data may exist, there needs to be a way of presenting a complete landscape of these. Moreover, patients should be able to understand these automatically generated patterns and adjust them to more actionable items for everyday life scenarios. Partial understanding and addressing of these sensemaking challenges—EHR data organization and automatic pattern detection—may often result in formulating skewed impressions of the patient’s health, thus generating misconceptions that may threaten their well-being. Previous research has provided some insights into alerting and organizing personal information, but more work is necessary for applying these concepts to EHR data.

A more comprehensive and engaging approach to pattern detection and alerting the patient to ongoing issues can be found in self-monitoring applications. Notifications for meeting goals [26] or recommendations [27] guide the user to take action for health improvement based on the patterns in the data streams coming from mobile sensors, manually entered self-assessment observations, and instrument measurements. However, such sophisticated pattern detection and recommendations can be very challenging to determine and compose owing to the sparsity and incompleteness of EHR data [28]. In addition, alerting and directing the patient based on pattern detection in EHR data poses certain risks as messages need to be framed differently than in a clinical setting. This framing must leave no room for misinterpretation by a layperson and must prevent harm at the same time [29,30]. Borrowing from existing approaches and relying on the literature, special attention should be paid to the formatting and presentation of alerts based on EHR data.

Furthermore, an opportunity to overcome the data organization shortcomings in existing applications lies in the format that is used to make the EHR data available to patients. Fast Healthcare Interoperability Resources (FHIR) is an EHR data interoperability standard that has dedicated resources for each data type that can be encountered in medical practice, such as conditions, procedures, and laboratory test results [31]. Therefore, the patient’s EHR data from multiple providers could be modeled as a set of FHIR resources (ie, records that come from multiple contributors). This setup allows us to draw analogies from existing work that focused on organizing web search results [32], relevant excerpts from web pages [33], brainstorming results from large groups [34], and pictures from the web [35]. In the spirit of the sensemaking data-framing theory, the general idea behind this work is that there exist some individual pieces of facts (ie, evidence) that need to be brought together and schematized to surface some actionable meaning. Related to this, researchers identified collections of various files (analogous to records in our case) as the most convenient and well-received way to organize data for nonexperts [36,37]. Therefore, efforts should be made to leverage this opportunity and enable personalized EHR data organization for patients.

### Objectives

In summary, there are still open questions regarding (1) how patients would welcome and engage with artificial intelligence (AI) recommendations based on patterns in their EHR data and (2) how and why patients would want to organize their EHR data to support their sensemaking.

To address these gaps, we asked the following research questions (RQs): (1) *How can we meaningfully surface automatically detected patterns for making sense of EHR data from multiple providers? In what forms and to which extent would patients want to receive such automatic support?* (RQ 1); (2) *How can we support the organization of EHR data to suit the patient’s needs? Why and how would patients want to organize their EHR data?* (RQ 2); and (3) *How can these sensemaking support improvements potentially benefit the patients?* (RQ 3)*.*

To answer these RQs, we conducted an inquisitive research-through-design study with 14 participants. Presented in the context of a cutting-edge application with a strong emphasis on independent EHR data sensemaking, called Discovery, we provided patients with high-level design mock-ups. These mock-ups demonstrated the capability for personalized transformation of EHR data into problem-based structures: (1) system-generated alerts, where the system mines the EHR data to identify data patterns that reflect potential problems and offers recommendations, and (2) manually created collections of medical records based on health issues. By presenting the participants with such capabilities to organize and look at their EHR data based on health issues or potential health problems, we unlocked their capability for answering our RQs and conducted an inquiry into understanding how these new capabilities will affect patients’ engagement with their EHR data.

In the remainder of this paper, we present our methods and findings and provide a discussion around their interpretation and contribution. Finally, we offer some design implications and conclusions.

## Methods

### Overview

We conducted an inquisitive research-through-design study. This study was centered on mock-ups for 2 novel features: *Alerts* and *Collections*. The primary goal of these mock-ups was to instantiate and concretize the complex concepts that these features rely on. At its core, our approach has a research-through-design direction [38] as we believe that only after looking at these mock-ups would the study participants be able to more clearly envision our ideas and contribute to answering our RQs, which would otherwise be impossible, poorly articulated, or vague. In contrast, our work also has elements of design as inquiry. Similar to the work of Rosner [39] that introduced a new way to navigate maps, we introduced new ways for patients to navigate their medical records. Analogous to the approach by Rosner [39], we left it more open-ended in terms of the concrete needs we want to address with our design. We decided to let the study participants be inspired by the novel features and tell us more about what they believe could be achieved with those features or what improvements could be made for meeting needs that are currently not or only partially addressed by our design.

In our study, and to explore the design space more broadly by invoking feedback from a variety of potential users, we used healthy participants and participants with acute and chronic illnesses who had previously evaluated Discovery. Through Discovery, these participants were already exposed to the novel idea of making sense of medical records from multiple providers and had the necessary experience to be able to think about potential improvements in the sensemaking process.

### Description of Discovery

Discovery is an open-source patient-facing sensemaking support web application for EHR data that come from multiple providers [21,40]. In this context, a provider could be any institution that provides care, such as hospitals, clinics, or private practices. Discovery works with a subset of the structured EHR data from the US core standard [41], disregarding free-text clinical notes. In its current version, it only focuses on helping patients find records relevant to their questions. However, despite providing multiple specialized views to look at the data, convenient layouts, and visualizations, this process is predominantly manual. In addition, there is no support for organizing the relevant records for a given question. For a more detailed description of Discovery, please refer to the study by Nakikj et al [9], where the authors explain the data access and its features and usability.

The key reason why we used Discovery for our study is its convenient data model based on FHIR resources that we refer to as records. At the highest level, we have the record types (eg, conditions, encounters, and immunizations). For each of these, there are record subtypes, for example, immunizations (human papillomavirus quadrivalent; influenza, seasonal, injectable, and preservative free; meningococcal conjugate vaccine; and tetanus, diphtheria, and pertussis vaccine). Each record subtype can have one or more individual records that were created at different points in time. The provenance of the record is labeled with the name of the provider (institution) that they come from. At this stage of the design, Discovery does not offer more details about which particular clinician created the record. The records, being atomic and distinguishable data structures in Discovery, will be the subject of eliciting data frames through automatic pattern detection and manual organization in our advanced sensemaking features.

We want to note that the ultimate goal of a patient-centered sensemaking support tool should always consider improving the patient-provider communication. In that spirit, we hope that the features that we are gradually trying to introduce will benefit patients to obtain a better grasp of their medical records and, in doing so, have more informed and grounded communication with their providers. However, the designs we explore in this particular study and the RQs are mostly focused on supporting patients’ sensemaking rather than patient-provider communication.

### Study Design

#### Participants

We included 14 participants who had evaluated Discovery in a previous study. They were recruited through advertisements on Craigslist. We balanced the sample so that we had a variety of participants with respect to age, gender, and medical history. The participants had to meet the following eligibility criteria: being an adult fluent in English with a working laptop or desktop computer (with a screen size of ≥13 in), stable internet connection, normal vision or well-corrected vision with glasses or lenses, no color blindness, and medical records with one or multiple providers or institutions (hospitals or private clinical practices).

#### Materials

##### Overview

We created high-level mock-ups to roughly concretize our complex and abstract ideas for pattern detection in EHR data (*Alerts*) and the organization of those data (*Collections*). Both *Alerts* and *Collections* have the ability to transform EHR data into data frames for more efficient sensemaking—the first one being an automatic approach and the second one being manual. The data frame for an alert is centered on a meaningful pattern from the EHR data that is automatically detected by the application and is not modifiable by the patient. In that sense, the alert already completes a fundamental sensemaking task for the patient—finding a pattern in the data. In addition, the alert has a visualization of the pattern and explanation for better understanding and conveying meaning. However, the patient still has to perform further sensemaking by considering other alerts that have also been produced by the system. In contrast, the patient manually creates a data frame by putting relevant medical records in a collection. The meaningful data pattern (or patterns) within this frame is yet to be found by the patient as the collection evolves and matures. In contrast to the alerts, the patient is able to edit the contents of the collections by adding or removing records and textual notes. Similar to the assembly of alerts, the patient can rely on the collections they have created for more comprehensive sensemaking of their health situation and medical history.

To secure an inquisitive approach, the level of detail in the mock-ups was just enough to anchor and stimulate the discussion in the desired direction and provoke brainstorming at the end of the session.

##### Alerts Feature

The *Alerts* should allow patients to have access to important and yet hidden patterns in their data that are nontrivial to detect or even unobvious to look for. Driven by well-established clinical guidelines and medical knowledge bases, the alerts should surface potential issues for patients and raise their awareness of possible upcoming problems that need to be addressed. It is important to note that the alerts in Discovery are not intended to replace physicians. Rather, they serve as an *advocate* for patients for matters that are otherwise unobservable for them or even for their physicians. This type of advocacy is necessary because of the fragmented data across providers and poor capabilities for comprehensive, multi-provider data pattern detection in existing patient-facing applications and EHRs.

**Figure 1:**
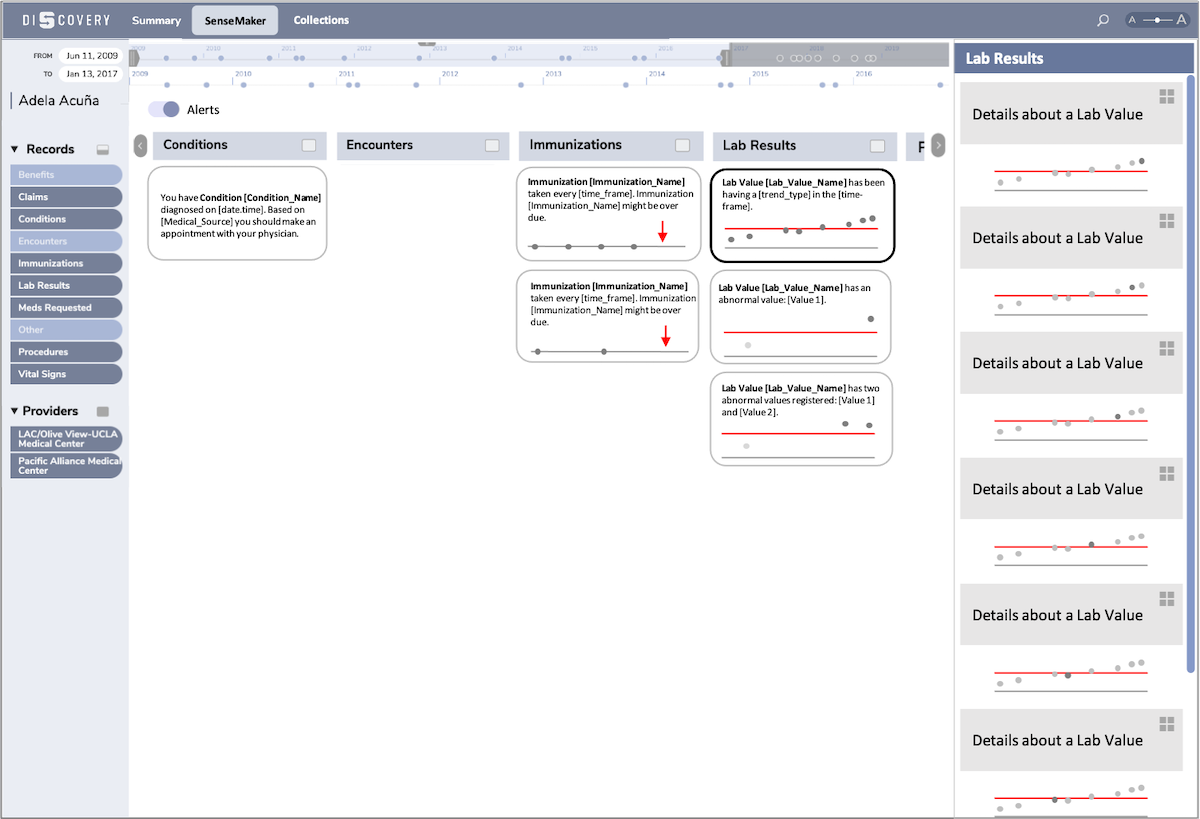
The high-level design mock-up for the *Alerts* feature. LAC: Los Angeles County; UCLA: University of California, Los Angeles.

In the mock-up in Figure 1, we present a possible iteration of the design for Discovery. Conceptually, the *SenseMaker* is the new view that unifies the functionalities of the current multiple views in Discovery and is the single place where the user goes to identify interesting patterns. Although how the *SenseMaker* looks is not relevant for this study, it was important to show that the user will be able to toggle between manual foraging for patterns and automatic pattern detection—the *Alerts* feature.

Our design was inspired by the success of clinical dashboards [42]. These dashboards typically display multiple trends in patients’ data coming from a variety of sources, with separate visualizations on a single screen [43]. They often use an AI agent that mines the data for patterns and is capable of making predictions [44,45]. Using markers or messages, they display alerts that draw the clinician’s attention to a potential issue or threat to the patient. Analogously but with appropriate modifications and simplifications, we put the alerts in a panel where they can immediately and transparently provide context to one another and take an active role in broader sensemaking activities, offering a novel approach to making sense of the EHR data for patients. The alerts are organized by record types and constructed based on triplets: a pattern identified in a single data variable (for simplicity), a *visualization* that presents the pattern, and the message that provides *explanation* and *recommendations* for actions. For our mock-ups, we used toy examples that were manually created and not based on any actual pattern detection or recommendation generation service, and we did not use clinical guidelines or medical knowledge bases for this purpose.

In Figure 1, the *Alerts* feature detects a pattern in the conditions and recommends that the patient should make a physician’s appointment soon; it then looks into the history of immunizations and realizes that 2 immunizations are overdue; and, finally, inspects the various laboratory test results and notices an upward trend in one of them (eg, cholesterol measurements), plus some values, different than what is considered normal range, that deserve further attention in 2 other laboratory test result variables (eg, blood glucose and glycated hemoglobin). After clicking on the alert with an upward trend in the laboratory test result, it is automatically shown in the *Details Panel* on the right. Here, there is a more detailed visualization and explanation of each of the laboratory test values individually.

##### Collections Feature

The *Collections* should allow patients to organize and annotate their medical records in a way that best suits their information needs. For example, a patient dealing with high blood pressure can create a “High Blood Pressure” collection and populate it with all their high blood pressure readings from clinical visits in the past, perhaps high BMI, or any other medical record relevant to the issue. They can add notes to the individual records for context of the measurements (eg, events from everyday life and behaviors), such as stress at work or eating large meals with alcohol. With the notes, they can also capture an insight surfaced from the collection, such as trends in the data or possible correlations among variables, for example, a relationship between high BMI and high blood pressure.

This form of data organization and insight retention capability is lacking in existing solutions. Consequently, it imposes repeated and tedious sifting through medical records in an attempt to identify the relevant ones over and over again to replicate inferences and recreate mental notes for current and frequent information needs. With the collections, in contrast, patients would be able to quickly look up and access information assembled for a particular ongoing issue and have an organized medical history, with insights, for later reflection and planning.

**Figure 2:**
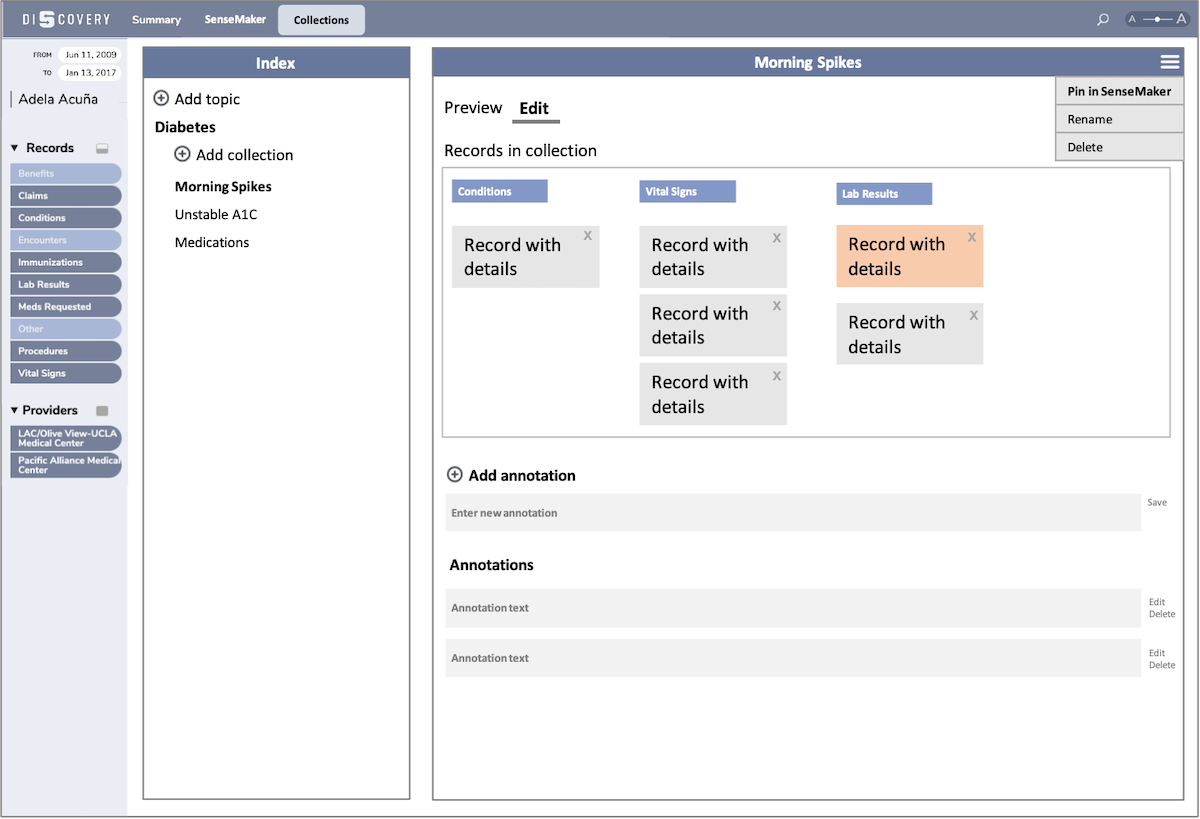
The high-level design mock-up for the *Collections* feature. LAC: Los Angeles County; UCLA: University of California, Los Angeles.

The key idea for the *Collections* feature is that, as the patient explores their data in the *SenseMaker*, they can add or remove records from the named collections using simple artifact marking mechanisms popular for assembling information on the web (saving a photo, ie, a pin in a Pinterest collection, or bookmarking a web page or an Instagram post) [35]. In the mock-up for the *Collections* view (Figure 2), there is a *Collection Index* and *Collection Inspector*. The *Collection Index* is a nested list of all collections that the patient created and allows for quick access to a particular collection. The *Collection Inspector* is the place where the patient inspects, modifies, and annotates the collection selected from the index. In the example shown in Figure 2, the user created a topic about diabetes. Within that topic, the user added 3 collections: morning spikes, unstable A_1C_, and medications. The first one, morning spikes, was selected for previewing or editing. The *Preview* only displays the records in the collection organized by record type together with the notes. In the high-level mock-up in Figure 2, we can see that the patient collected 1 condition, 3 relevant vital signs, and 2 laboratory test results. We can also see that they created 2 notes that, for example, captured measurements they made with additional context explanation. To modify the collection, the patient goes to the *Edit* tab. There, they can delete records or add, edit, or delete notes. In case the patient wants to add new records to the collection or simply continue exploring starting from a given collection, they can pin it to the *SenseMaker* and jump to that view.

#### Procedures

##### Overview

We took inspiration from the existing literature on research-through-design [38] and design as inquiry [39] to formulate our approach. We also explored ideas from design studies in the medical domain that aim to enrich the use cases, attitudes, design requirements, and functionalities of patient-facing digital tools to help us formulate the questions for the study participants [46,47]. We conducted a remote study using Zoom (Zoom Video Communications) meetings. In the 60-minute study session, the researcher shared the screen with the participant and displayed the corresponding mock-ups following a script or when the participant demanded it for reminding purposes or reference points.

We reused the demographic data to characterize the participants as digital health consumers from a previous Discovery study that they had already completed (Textbox 1). As the participants were already familiar with Discovery, the researcher only spent a little time introducing the new study and proceeded with the block dedicated to obtaining feedback on the *Alerts* (Textbox 2), followed by the analogous block for the *Collections* (Textbox 2). The study session concluded with brainstorming on new sensemaking support features (Textbox 2) inspired by the previous 2 blocks.

**The questions for understanding the user as a digital health consumer.**

- What is your age?
- How would you describe your medical history—have you been seeing physicians a lot or not?
  Do you have any chronic conditions—anything that makes you monitor your health more closely and have more frequent physician’s visits over longer period of time?
- How many different providers and institutions have medical information about you?
- How hard would you say it is to keep track of your medical information from those providers and institution?
  What would be the biggest barrier for doing that?
- How comfortable are you with technology?
  Do you currently use any devices to keep track and make sense of your health and medical information? What do you like and dislike about them?

**The semistructured interview for advanced sensemaking features inspired by the mock-ups: *Alerts*, *Collections*, and brainstorming for new sensemaking support features.**

***Alerts* feedback (20 min)**

- Sensemaking driven by an artificial intelligence (AI) agent (10 min)
  How would you feel if there was some AI agent behind the scenes looking for patterns in your data and alerting you if it finds something interesting?
  How would you see your autonomy if an AI agent drives the making sense of your data instead of you?
- Feedback on the *Alerts* after mock-up presentation (10 min)
  What is your opinion of the *Alerts* feature I just presented to you?
  What are some things you liked and what is something you didn’t like?
  What are some changes or improvements you’d like to see?
  Do you have any worries about how those patterns are detected? Do you worry about their reliability or missing some of the important ones?
  When would you see the most potential for this feature to help you?

***Collections* feedback (25 min)**

- Data organization and reflecting on previous sensemaking (10 min)
  Currently, Discovery doesn’t support any organization of the records you identify as relevant for your questions during making sense of your data. However, we would like to support that. How would you like to be able to organize your medical data as you are finding answers to your questions?
  How would you like to be able to organize your medical data on top of what Discovery supports now for data exploration?
  What are some ways in which you would like to be able to reflect on previous explorations of your medical data?
- Preparing for clinical visits (5 min)
  Discovery is also meant to help you better prepare for your upcoming clinical visits. What would help you quickly reflect and remind yourself about the key points you want to cover in the visit and the evidence in support of that?
- Feedback on the *Collections* after mock-up presentation (10 min)
  What is your opinion of the *Collections?*
  What are some things you liked and what is something you didn’t like?
  What are some changes or improvements you’d like to see?
  When would you see the most potential for this feature to help you?

**Brainstorming for new sensemaking features (10 min)**

- Future improvements of Discovery (10 min)
  Now that you have a better sense of where Discovery wants to move in the future, do you have a better idea of what are some features you would like to see, but are still not there?

##### Alerts Feedback

At the beginning of this block, the participants were asked an open-ended question related to how they felt about having an AI agent going through their EHR data, finding interesting patterns, and alerting the participants about them, thus taking over the driving seat in the sensemaking process (*Sensemaking driven by an AI agent* in Textbox 2). This was done before showing the *Alerts* feature mock-up to obtain an unbiased answer. Afterward, we introduced the mock-up to be more concrete about the previous idea and inspire talking points in the semistructured feedback discussion (*Feedback on the Alerts after mock-up presentation* in Textbox 2).

##### Collections Feedback

A similar approach was taken for the *Collections* feature. We first started an open-ended discussion around the participants’ purpose and desire to organize and structure the relevant records they identified during the sensemaking process (*Data organization and reflecting on previous sensemaking* in Textbox 2). Later, this was geared toward reflection and planning, especially for clinical visits (*Preparing for clinical visits* in Textbox 2). After that, we were more concrete by showing the mock-up for the *Collections*. Participants provided feedback on what they saw and how they envisioned this feature for their personal use (*Feedback on the Collections after mock-up presentation* in Textbox 2).

##### Brainstorming for New Sensemaking Support Features

After completing the 2 blocks for the *Alerts* and *Collections*, participants were asked to provide unrestricted feedback on potential improvements and suggest new advanced sensemaking support features (Textbox 2).

#### Data Collection

The study session was audio recorded and transcribed using a professional service [48]. Written notes were also taken during the session and combined with the transcripts for analysis.

### Data Analysis

The combined transcripts and in-study notes were thematically analyzed [49]. We took the reflexive thematic analysis approach [50]—one that allows for a more organic and flexible coding process. In this approach, there is no code book, and coding can be performed by one or more researchers, where the process is framed as a collaboration rather than reaching a consensus. The codes can evolve as the analysis progresses and are ultimately grouped into themes that convey meaning and insights regarding the subject of the investigation. In our particular case, the thematic analysis involved starting by open coding the textual data by the first author of the paper; the emerging categories were discussed and reconciled in a meeting with the second and last authors. Consequently, we surfaced the needs and boundaries of the participants for automated pattern detection and organization of their EHR data as well as the most important points in the perceptions, desired improvements, and intended use of the advanced sensemaking support features—*Alerts* and *Collections*. These categories were validated for revealing insightful themes in a group meeting with other researchers unfamiliar with Discovery and modified according to the feedback to produce the final taxonomy for the results.

### Ethics Approval

We obtained approval from our Harvard Faculty of Medicine institutional review board office to conduct this study (protocol number IRB20-1757).

### Informed Consent and Compensation

Each of the participants signed a consent form to take part in the study and was compensated with a US $20 Amazon gift card.

## Results

### Participants’ Characteristics

The participants ranged from those who considered themselves healthy (6/14, 43%), those who had episodes of acute conditions (4/14, 29%), and those who had to manage one or more chronic diseases (8/14, 57%). The age range of the participants was from 20 to 53 (mean 33.43, SD 10.39; median 30) years. We included 43% (6/14) male participants and 57% (8/14) female participants in the study. All participants (14/14, 100%) had a high school education, with most (10/14, 71%) having a college degree. Only a few participants (3/14, 21%) had professions that involved data analytics.

Some participants (2/14, 14%) had very few medical records with 1 or 2 providers, whereas others (7/14, 50%) had an abundance scattered among multiple providers, from 5 or 6 up to a dozen. The rest of the participants (5/14, 36%) had records in between these 2 ranges. The participants who had rare encounters with their few providers generally found that the patient portals were useful and met their very basic needs. In contrast, those who had a lot of highly fragmented data across many providers found the experience very frustrating. Remembering how the portals worked and manually pulling data together to prepare for clinical visits or just to understand their health status was reported to be very cognitively demanding and laborious. All but 1 participant (13/14, 93%) declared being very comfortable with technology and using it on a daily basis. Most participants (11/14, 79%) had some experience tracking their health data, for which they used basic applications for running or step counts. However, none of the participants had previous experience interacting with AI agents for making sense of their medical data, nor had they ever had a chance to organize their digital medical records based on their needs within a patient portal or other application.

We classified our findings into 2 broad topics: *Alerts in the process of EHR data sensemaking* (RQ 1 and RQ 3) and *Collections as tools for EHR data organization* (RQ 2 and RQ 3)*.* For the *Alerts*, we identified the following themes—*implications on health attitudes, determining utilization potential, classification and appropriate dosing, keeping track of alerts,* and *communication with providers*—and, for the *Collections*, we identified the following themes—*use cases for the collections, generating and organizing the collections, enriching collections with PGD,* and *communication with providers*.

In the remainder of the *Results* section, we will report on these themes and provide 19 quotes from 10 different participants, labeled P1 to P14.

### Alerts in the Process of EHR Data Sensemaking

#### Overview

The vast majority of participants (12/14, 86%) were open to having the *Alerts* in their sensemaking features arsenal. In contrast, very few (2/14, 14%) wanted to stay away from them, stating that they were taking over the task that only a physician is equipped to perform:

This actually looks really fantastic. I feel like having these alerts, just being told what you are looking out for would be useful to me. I feel like, I don’t know, I tend to forget these things and especially looking to your...just sort of these reminders so that an immunization that might be overdue, telling me that I should make an appointment with my physician for this condition I’ve been diagnosed with. I think that these would be useful to me and I can see this sort of thing just ensuring that I am going regularly to the doctor and getting preventative care done. I think also having these, I guess, abnormalities and trends in my lab work pointed out too, that’s really useful, and I think that this is stuff that I wouldn’t necessarily know to look for on my own if I were just going through my data. The way these alerts are, it does seem like they are just worded softly enough but they still get the message across well.

P11

The idea that this software would be what would be informing me about this rather than a professional, it seems like I’d have a hard time putting my faith in it...To me, that’s the doctor’s job, that there is all this data to filter through. It’s overwhelming, so you need a professional who understands your priorities and their priorities...My fear is that because we’re relying here on AI to find likely patterns, we’re missing the nuance.

P9

Trust and actionability of the alerts determined how much power they would have in sensemaking, and descriptors such as topic, urgency, currency, and the sentiment of the alert determined the priority and the attitude toward it. Despite the potential usefulness of the alerts, concerns were raised about how to determine the appropriate amount and frequency. Furthermore, the history of alerts was perceived to have the potential to be transformed into a knowledge repository for dealing with health issues. Finally, the alerts were expected to support context- and evidence-based patient-provider communication and establish shared mental models between the 2. A more detailed report of these findings is presented in the following subsections.

#### Implications for Health Attitudes

Participants reported several possible implications of the *Alerts* feature on their health attitude. They anticipated that the alerts could raise their health awareness and stimulate their proactivity but also increase their anxiety. The participants mostly agreed that the alerts could offer a comprehensive landscape of the ongoing or upcoming issues, thus increasing their awareness of their current health status or the potential directions in which it may move. Furthermore, participants welcomed the *Alerts* feature as a device to stimulate their proactivity, and the alerts were perceived as a powerful nudge to prevent potential exacerbated inconveniences or significant deteriorations. However, a number of participants (4/14, 29%) were mainly concerned that this feature would keep showing numerous alerts in amounts very hard or impossible to keep up with, thus creating a possibility for anxiety.

#### Determining Use Potential

In total, 2 factors, trust and actionability, influenced how much participants would use the alerts.

Participants who were open to using the *Alerts* feature expressed variable levels of trust in the pattern detection algorithms and the recommendations based on them. Although some (5/14, 36%) viewed this form of AI support as an opportunity to obtain an approximate and comprehensive overview of their health status, others (2/14, 14%) felt that this approach could be very unreliable and were extremely cautious about the extent to which they would rely on it:

I would always check it. There’s so many things that you don’t rely on just one. Myself, I have that mentality, but I’m not sure. You don’t just rely on the system entirely ever, but it’s nice to see what it catches that you weren’t able, for example, to catch, or what other things that didn’t catch that caught your attention by analyzing the data yourself, by looking at the data yourself.

P2

Participants also wanted to know why certain patterns matter, what are the descriptions and explanations of those patterns, and what is the authority behind them—well-established clinical guidelines, hospital knowledge bases, or conclusions drawn from a large number of Discovery users. In addition, they valued the visualizations as an important complement to the verbal explanations:

If we’re assuming that this app is only being used in the US, we can say, “According to the top hospitals, research shows that the reasons why you should get your flu shots every year is because blah, blah, blah, because your body might use immunity to the flu over time, because it takes your body X amount of weeks to actually process and absorb the vaccine,” that type of thing.

P6

Participants put a strong emphasis on the way the recommendations were formulated—what actions they could take based on the alerts that were safe and good for them. They mostly wanted clear, unambiguous messages that moved away from a strict clinical recommendation, something that only the physician should be responsible for. However, “mild” recommendations or declarations about the status of health that orient the patient and give them a sense of direction were welcomed for the most part:

Because there’s one thing it marks an upward trend, but there’s an upward trend that’s unsafe and an upward trend that’s not as bad, so it may have not reached an unhealthy level, but it’s enough of an upward trend that you might want to keep an eye on it...[For example] I’m noticing that your cholesterol has an upward trajectory but you’re still in the normal numbers, so this is something you should talk to a doctor about but it doesn’t seem as bad...

P3

#### Classification and Appropriate Dosing

The participants felt overwhelmed by the potential number of alerts and desired additional capabilities of structuring and organizing the alerts by topic, urgency, currency, and sentiment.

Some participants (3/14, 21%) wanted to see the alerts organized by disease or condition. They felt that this organization would make it easier for them to prioritize the numerous alerts and tackle them in a more methodological way. Several participants (4/14, 29%) noted that some alerts may need immediate attention and others could be taken care of at later times, wishing for an easy distinction between the 2:

Maybe like you were saying if something’s urgent, like I get a lab result back and it says that I’m pre-diabetic, that would be, I think, urgent, as opposed to getting a lab back, which I have, that I had low vitamin D, so I started taking vitamin D. So that would be maybe a medium alert.

P8

A couple of participants (2/14, 14%) pointed out that, over time, some urgent alerts could become outdated or have been already taken care of and expressed the need for keeping track of the alerts’ currency. Several participants (3/14, 21%) shared the notion that the alerts should not exclusively stress negative trends or focus only on undesired outcomes but also point out when the patient is doing well in certain aspects of their health. According to the participants, this approach should contribute to avoiding perpetual worrying and depressive sentiments associated with the *Alerts* feature and also provide a sense of encouragement and accomplishment, when applicable:

It were great to see a pop-up or email saying, “Great.” It’s like, “Your cholesterol has gone down. Your BMI, like you said, is going down. You have a lower dosage of medication. Keep it up.”

P14

Although most of the participants (12/14, 86%) liked the idea of the alerts, they expressed worries about being overwhelmed by a potentially huge number of them. To avoid this, they wanted to be able to prioritize the alerts based on the previously covered classifications (from a previous paragraph) and determine when to look at them: pushed as detected; upon opening the application or only during exploration; or once or multiple times a day, week, or month.

#### Keeping Track of the Alerts

Almost half (6/14, 43%) of the participants perceived the *Alerts* feature as a log of open issues that need to be taken care of. However, they also acknowledged that taking care of such issues is not a straightforward task and might involve multiple steps. For these reasons, they wanted some capabilities to track the progress toward resolving the open issues, adding notes or tags. However, a couple of participants (2/14, 14%) raised concerns about who should be providing those tags—the patient or the physician.

#### Communication With Providers

Patients felt empowered by the *Alerts* through which they could potentially message their providers in the future, securing enough context and evidence with the click of a button. In addition, some expected that, as there is a notion of system-validated alert, the provider would more likely pay attention to that message and respond. In many cases, participants felt that they would need professional help to assess how urgent or important a certain alert was and further validate the recommendations it provided:

I’d like to have to be able to contact my doctor if I see an alert, just something that it hasn’t come up before, something that I haven’t discussed with her before. So, I would like to send that information to her saying, “Discovery is picking up on this. Is it something I should worry about?”

P2

However, some participants (3/14, 21%) raised the concern that patients might start “pinging” their providers for every single alert they encountered, overwhelming the physician to the point where they start ignoring their messages, ultimately hindering their relationship.

Interestingly, a couple of participants (2/14, 14%) laid out a flipped scenario of using the alerts for communication—instead of the patient making sense of their importance and deciding what actions to take, it should be the physician who performs that curation and contacts the patient first:

The way I see it is that the doctor would get an alert, these trends. All this stuff is fine for the doctor to get. Doctor goes through and says, “Oh, yeah. Weight is going down. That’s okay. We expected that. Immunizations, yeah. I should have the secretary call.” Or, “Let me click and invite them to an appointment.” Something like that, but the doctor’s got to be in the loop, and I think it goes to the doctor first.

P9

Similar to the use of the alerts for messaging with their providers, participants saw their value for in-person communication during clinical visits. They regarded the alerts as on-the-spot conversation drivers that provided satisfactory context and evidence.

### Collections as Tools for Organizing EHR Data

#### Overview

Participants embraced the notion of grouping records relevant to a particular topic or issue under named collections and recognized how they could help in raising awareness, reflection, and health tracking. However, they wished for more flexibility in generating, enriching, and organizing the collections, as well as features that would support using them for efficient patient-provider communication:

I feel like I would just make topics just around this chronic conditions of mine. I think I would use it to keep track of say, lab work especially over time, but also how that might line up with say, even my vital signs or...and also just put my doctor visits in there to see just so I can have a complete image of a particular condition over time in all of its different aspects.

P11

A more detailed report of these findings is presented in the following subsections.

#### Use Cases for the Collections

Participants recognized a wide range of use cases for the *Collections* feature, such as quick access to relevant records, reflection on and awareness of their health, and tracking their health and journaling their diseases.

First, they suggested that they would use the collections for quickly looking up the records related to a pressing issue or other questions that frequented their minds. A mapping between frequent information needs and relevant records was one of the major benefits of the collections:

Yes. Exactly [access relevant information fast]. So, next time she logs in, then she just have to know where to go and click and then she will see everything and then she can add some annotation?

P4

Second, they perceived the collections as a reflection vehicle for reviewing their health status and medical history by having all sorts of issues and topics well organized and documented. This also provided a repository for raising awareness of their current health status that can be easily accessed on demand:

I think that would be useful. I think that’d be really...that’d be interesting at the very least to see how my condition has evolved and also how my thoughts and records of this condition have evolved.

P11

According to the participants, the collections can also provide a great platform for tracking their health by inserting free-text or structured notes with concrete values. They thought that these notes could be for an individual record from the collection or the entire collection. Similarly, the participants saw the potential to journal their emotions and daily experiences with the diseases on a more elaborate level using the free-text notes and potentially capture important subtleties for understanding the effects of a treatment or disease progression or tracing back events that might have affected certain outcomes:

Just to let him [the physician] know that there’s a pattern. If there’s a certain pattern of food I’m eating and then I’m having these asthma attacks. Is it because I’m in a certain environment? It is the time of year? So, I would just track what happened, because what leads up to hours prior can determine. And it’s hard to think about it, so it’s nice to have. Think about it in long term, so it’s nice to write it down and always look at it. Because then it makes more sense.

P7

#### Generating and Organizing the Collections

Participants liked the possibility of grouping and organizing their records. However, some (5/14, 36%) complained that the process was extremely manual, which made it hard to determine which records to look for and how to assess their relevance. Therefore, they wished for a level of automation in identifying the relevant records for an issue or topic. Suggestions about having prepopulated collections with records and allowing the user to modify them as necessary were offered as one of the solutions:

I think a template would be extremely useful for these things and would make me a lot more likely to use the Collections then rather than having to go and populate it myself. I trust that selecting something for my chronic condition might include categories that I forget about myself, so it would make it just a lot easier and a lot faster.

P11

The majority of the participants (9/14, 64%) thought that having more than one level of nesting would benefit the organization of the collections. This was primarily due to the complexity of certain diseases and conditions and the need to branch them further for higher granularity to tackle problems more specifically. In addition, some participants (4/14, 29%) wished to be able to link the collections. First, they believed that this is important for the reusability of the collections—some diseases might share relevant records, and thus, separate collections related to such diseases can extend a link to an existing collection that holds those records. Furthermore, because of associations between different diseases or aspects of them, they wanted to establish relationships between different collections. Finally, they wanted to capture the evolution of diseases, for example, where one disease stemmed from another:

I think I’d be curious how much control I have as a user over setting and sort of manipulating a collection versus the Discovery system itself. For instance I can think of conditions and events I have that over time have become interrelated even though they started maybe as one off things especially when I was younger or a child but have sort of morphed as adulthood has happened into semi chronic conditions. And I sort of create a nesting effect of those and create like parent-child relationship with pins or how that grouping is happening.

P13

#### Enriching Collections With PGD

Although the patients felt good about organizing records in collections, they showed reservations about the records’ comprehensiveness. They said that much of the medical events happen outside of clinical visits and deserve to be captured easily and on a regular basis:

I would just give a brief explanation. Like what happened. Like, “Oh, I had an asthma attack because I was with something that I was allergic to.” Or, “I ran too much.” I’d just give a little detailed description that the hospital wouldn’t give...Where if something happened like, “Hey, they screwed up the vital signs even though they’re on here, they weren’t accurate. Because the pulse ox strokes and they forgot to change it.” I don’t know. Just little things...Also, just to jog my memory of, “Hey, this happened when I tried to eat shrimp.”

P7

Despite the power of free-text notes, most participants (11/14, 79%) proposed entering more structured text to capture quantifiable observations based on third-party devices. These measurements, on occasion, needed to be summarized before being entered into the application. The participants also asked for interfacing to health monitoring and tracking devices that would result in a more continuous and automatic provision of data.

Some participants (3/14, 21%) went so far as to propose a special type of record for PGD that would complement the other record types. These records would be there to store not only observations and concrete quantified values but also life events, which more often than not are causes for health to take certain directions:

Well, I think you could have a record type called “patient events.”...Yeah. So I could mark, “Oh, here’s when I got married.” Then, “Oh, look. Ever since I got married, my blood pressure has been up.”

P9

#### Communication With Providers

The collections were perceived as a powerful tool in preparation for clinical visits and providing contextualized and evidence-based communication with the provider.

Having the capability to organize their data in collections before clinical visits would give participants the power to prepare the topics they wanted to cover and ask the right questions without forgetting:

I think that sounds great [collections]. I think, especially in the sense of if you have an appointment it would be nice to be able to show a doctor, I want to ask you a question about this, and I think just being...having being able to log certain things on an app is helpful. But I think I even see it just in terms of being able to ask people questions. I think that’s really helpful, because I think it could empower people too...they’re looking at their records in an easy to access way and they might be able to ask questions that they wouldn’t have thought of before.

P3

The collections were perceived to efficiently familiarize the physician with the data related to the patient’s questions during the visit and establish a shared mental model of what the priorities for the patient are and why, as well as share the progress that has been achieved since the last visit:

I like that. I like the idea [collections]. Yeah. Because sometimes it’s like when I have a doctor’s visit, a dermatologist, for example. Like, “Oh, I use this. You suggested that I do this.” Just to have all that information of whatever prescription creams and stuff she gave me last time, and how it has worked. I don’t know. I think it would be nice to have it as a backup, especially for people that have complicated conditions, I would say.

P2

## Discussion

### Novelty, Methodology and Principal Findings

To the best of our knowledge, this study represents the first attempt to understand how to apply principles of the data frame sensemaking theory to support patients’ sensemaking of their EHR data from multiple providers. We explored 2 concepts related to enabling the transformation of EHR data into “frames.” The first one was about automatic extraction of meaningful patterns from the EHR data—*Alerts*. The second one was about manual organization of the EHR data around health issues—*Collections*, within which patterns could be independently observed by the patient. This study showed great interest in these novel ideas but also demonstrated that there is still a long path to carefully walk for producing designs applicable to real-life scenarios.

With our research-through-design approach, we could obtain insights about patients’ needs and preferences regarding new ways of engaging with their EHR data. The richness and reliability of the answers to our RQs related to usefulness, representations, and interactions with organized EHR data were heavily conditioned by the existence of the mock-ups. By presenting mock-ups of complex and novel features such as the *Collections* and *Alerts*, we unveiled a new frontier in patients’ conceptualization and sensemaking of their EHR data. Suddenly, patients could more concretely envision seeing their data not as a list of records ordered by time, type, or care provider but organized in a way that suits their information needs using the collections and reflects the potentially emerging health problems that deserve attention based on the system-generated alerts. This transformation from a rather crude dissection of the EHR data by type, time, and care provider to a more granular and problem-oriented view is a significant shift that became reasonably tangible and graspable to the participants in the presence of the mock-ups. Furthermore, we addressed a broader need for efficient and reliable sensemaking by introducing capabilities to transform the outlook of the EHR data into frames: manually—in collections—and system-driven—in alerts. Using this approach, we also enabled patients to retain and reuse the sensemaking work performed on their EHR data. By presenting these powerful capabilities through design mock-ups, we were able to inquire into patients’ needs, uses, and preferences related to problem-organized EHR data. We observed reports of various use cases that we could not previously envision as well as unanticipated functionalities such as strong emphasis on enriching the EHR data with PGD and bringing the physician in the loop as a supervisor and validator of their sensemaking work.

To conclude, our approach provided original contributions to the biomedical informatics and human-computer interaction fields. First, it validated the design assumption that patients want to have their data organized based on their information needs regarding current health issues and ongoing medical problems. Second, the study emphasized the importance of creating an ecosystem of EHR data and PGD that live under the same umbrella and complement each other within the confines of designated, problem-based collections. Third, the study pointed out the need for automatic support in data organization, either through automatic building of the collections or automatically detecting patterns in the data that carry some health-related meaning, good (progress) or bad (deterioration). Finally, the study indicated the importance of the physician’s role as a supervisor, validator, and editor of the sensemaking work that was performed by the patient manually or assisted by the system. We believe that these findings shed a new light on the way patients want to engage with their EHR data and open new horizons for further exploration of how to address their needs.

### Interpretation of the Results and Contributions

This work provides the following contributions to the fields of human-computer interaction and biomedical informatics: (1) *user needs and features for automated pattern detection in the EHR data from multiple providers—Alerts*; (2) *user needs and features for supporting organization and schematization of EHR data—Collections*; and (3) *design implications for improving the Alerts and Collections, enriching the PGD around them, and using these 2 new concepts in patient-provider communication.*

Most of the participants wanted well-crafted, contextualized, and pattern-based recommendations (ie, alerts that are taxonomized and prioritized for stimulating health proactivity and securing safety and actionability). They wanted the alerts to reflect not only threats of negative outcomes but also positive developments. By introducing annotations to the alerts, the participants also saw them as a platform for tracking progress in dealing with various health issues and a knowledge base for how to face similar challenges if they arise. Furthermore, the collections were regarded as a powerful tool for awareness, reflection, and planning. However, more structure within the collections, linking between the collections, and automatically generated collections were required. Participants stated that they would use the collection notes to log feelings, daily observations, and measurements, thus contributing to health tracking and disease journaling, but requested more variety in the formats for inputting their data. Finally, both the *Alerts* and *Collections* were perceived as a great opportunity for evidence-based communication with providers and tools for establishing a shared mental model of priorities for treatment and problem-solving.

In the attempt to propose a new way of supporting the sensemaking of medical records, we avoided the traditional user-centered design approach. This common approach typically focuses on a particular patient cohort and offers a design that meets their previously explored needs. In contrast, we decided to explore what a wider audience might expect when offered to engage with the capability to create collections of their own EHR data and see alerts based on automatically recognized data patterns. As this is a radically new approach to supporting sensemaking, we wanted to use our designs as tools for inquiring about use cases and needs rather than collecting feedback for improving a design based on previously narrowly defined and thoroughly researched user needs. That said, our designs were still well grounded in the existing literature on patients’ sensemaking of health data and our previous work. However, our designs were intentionally tailored to put emphasis on exploiting the fact that, once patients see novel features, they might start using them in unpredicted ways and for a variety of unaccounted purposes. For these reasons, we used mock-ups that were rough, provocative, and less refined but designed to let the study participants’ imagination fill in the gaps as they imagined scaffolded, personal experiences.

This approach enabled us to engage the participants in the designs by addressing broader needs that target almost anybody but then give them the opportunity to envision more specific, more personal needs and use cases. For example, by allowing data to be pulled from different providers, we address the fragmentation of the medical data for the patients—patients who start seeing a new specialist or move to a different city can benefit from that. In addition, by allowing for the organization of the data in collections, we enable more tailored engagement with the data based on issues that might benefit those dealing with multiple conditions or those who have a rich medical history. Furthermore, by allowing for alerts based on the patterns in the EHR data assembled from multiple providers, we assist in raising awareness of potential current and upcoming problems even for those who might consider themselves healthy or on top of their disease management. Although these needs are concrete, they are still broad. Given the complexity of health and medical knowledge, we wanted to dig deeper and unpack many other potential use cases and needs inspired by our new concepts of looking at EHR data. To this point, our results revealed key insights that we could not account for upfront. For example, the alerts were perceived as health status descriptors, desired to present potential deterioration but also improvements in health. Furthermore, annotations were required to keep track of issue resolution and contextualization for further use of the alerts as a knowledge base. Similarly, the collections were requested to have more internal structure and interlinking to respond to the complexity, relationships, and genesis of medical issues and diseases. In addition, various formats of PGD were needed to be more expressive in providing context for various health issues. Finally, there was a strong emphasis on the fact that the *Alerts* and *Collections* should be used in the communication with the provider, raising the perspective that these features should be designed as platforms for collaboration. We believe that the inquisitive design approach helped us uncover valuable needs and will enable us to tackle the design of the *Alerts* and *Collections* features in a more informed and traditional way further down the road—targeting particular patient groups with concrete sets of well-framed needs. For these reasons, the results of this study have the element of improving the scope of the design space and offering design directions rather than pushing a concrete design forward.

We found that most of the participants wanted some form of automatic pattern detection in their EHR data to support sensemaking and, similar to other studies, they needed well-crafted, pattern-based recommendations for establishing trust in the AI [51] and securing safety and actionability [27]. Previous work has put great emphasis on how to craft user-friendly presentations for explaining complex clinical topics [52,53] and how to deliver safe actions that patients should take based on data patterns [27,54]. Our study did not dig deep enough into these areas to provide notable findings. However, an interesting point we make is the need for a deeper context in the presentation of patterns beyond the typical reference to normal or values that are not within that range [55]. Participants wanted to have a better sense of how bad their health status actually was through the significance of the values in the pattern, the relationships between those values, and the possibilities to fluctuate in another better or worse category. In addition, and in contrast to traditional approaches that focus on notifying patients about the negative side of their health status and potential threats to their well-being [22], we found that the panel of alerts should also present the areas in which the patient is doing well.

Although there were several skeptical study participants, most (12/14, 86%) showed positive sentiments toward the alerts. However, the participants in the study were not formally familiarized with potential biases of the algorithms the *Alerts* feature could use in the future and might not have been aware of a variety of other limitations these algorithms can pose, such as working with sparse or incomplete data. In addition, the alerts mocked up in this study used a single variable (eg, laboratory test results); however, in reality, these will also include multiple variables (eg, medications and vital signs) and demographic information (eg, age, gender, and ethnicity). Therefore, the perceptions of the alerts may change as they become fully implemented and their limitations become more apparent. Although it appears that the alerts could be a powerful concept, we need to be aware of the potential bias in the predictions they make. The fairness of AI in health care [56] has been a popular research topic, and best efforts should be made to treat various demographics and cohorts with special attention. Our current design did not account for this as it is still in the early stages; however, tailoring the AI algorithms in the alerts to specific patient cohorts will be seriously considered for future iterations.

Aside from being transparent and objective about the AI that the system relies on, using understandable language for the patients throughout the interface and providing pervasive assistance for learning how to use the *Alerts* and *Collections* features will be very important factors for the adoption of these features. The language should be carefully designed to fully capture the meaning behind the offered interactions with the *Alerts* and *Collections* and provide patient-friendly terminology and explanations for clinically related information. Assistance in the form of tooltips and web-based, task-oriented video tutorials should also be available to patients. Although we are considering improvements in these aspects for our current designs, they were not subject to our investigation in this study.

Participants expressed a strong interest in contributing their own data to the *Alerts* and *Collections*. Previous work by Raj et al [10] provided insights into how caregivers of patients with diabetes make sense of their clinical data (eg, insulin dosage and carbohydrate intake) enriched with context (eg, location and exercise) to determine their effects on measurable outcomes (eg, blood glucose level). However, the clinical data used for this study do not carry the full meaning of the term “clinical,” which is usually associated with data that originate from a care provider institution such as a hospital or private clinic. In addition, the clinical data subject to the aforementioned study were very narrow and focused only on diabetes. In contrast, we focused our attention on clinical data in the traditional sense, the data that come from the EHR systems of the providers and are not limited to any particular disease. Therefore, our work extends the idea of enabling the sensemaking of contextualized clinical data by providing notes and annotations around EHR data.

We offer valuable insights into the value and use of PGD as enrichment to medical records, organized as alerts or collections. By adding annotations for progress toward resolving the issues represented in the alerts, the participants also saw the alerts as a repository of problem-solving knowledge that can accumulate over time. Furthermore, participants recognized an opportunity to use the notes for the individual records and the collections to log feelings, daily observations, and individual or summaries of measurements, thus contributing to health tracking and disease journaling. However, they also requested more variety in the formats for inputting their data and even suggested a separate type of record for those purposes. Reflecting on this, the idea of patients logging personal information in various formats, such as visuals, images, free text, or structured notes, for making sense of their health has been a long-standing research topic and is not novel [57-59]. However, doing so in the context of enriching the individual EHRs, patterns, and collections of them to support sensemaking around them and make those data structures more actionable in real-life scenarios is new and interesting.

On the basis of the results, the participants embraced the *Alerts* and *Collections* features as context and evidence providers as well as communication drivers that are capable of establishing shared mental models. This could probably be related to the challenges in patient-provider communication. These include difficulties in setting common ground or differences in identifying the problems and prioritizing them [60] and the need for patients to have some form of expert assistance in identifying and interpreting trends in their health data [10]. With respect to this, we should consider designing the *Alerts* and *Collections* as collaboration platforms for the patient and their physician rather than focusing exclusively on how those features can support sensemaking for the patient individually. Although the idea of a collaborative approach to treatment and diagnosis through messaging between the patient and the provider is not new [61], opening an opportunity that allows the patient to initiate communication with the click of a button in which there is a curated context and evidence already in place is relatively new to the medical domain.

However, although the *Alerts* and *Collections* features are promising tools for improving patient-provider communication, there are certain concerns related to how they can be used in the real-life workflow. First, there is the question of who creates the collections and who is able to modify them. It is conceivable that both the patient and physician can initiate a shareable collection and make edits or suggestions—the patient is the one who has much more time than the physician to dig through the data and knows their problems the best; the physician is the one with expert-level medical knowledge. Second, there is the question of who will curate the alerts. There needs to be an authority other than the AI agent—the physician, most likely—who can process the alerts and provide an interpretation of how reliable and important they are as well as what their priority is. It is conceivable that the physician can create an alert that was missed by the AI agent or override an existing one that they deem wrong, irrelevant, or inaccurate. Third, it should be noted that the designs in this study did not consider free-text clinical notes. Therefore, it remains to be further explored how these might affect patients’ organization of the EHR data and communication with their physicians. This is especially important to investigate as the lexicons that patients use typically differ from the ones in the clinical setting [62,63]. Moreover, different clinical roles—physicians (general practitioners and specialists) and nurses—may use different lexicons as well [64,65], and the notes they produce have different purposes in the overall care of the patient [66,67]. Similarly, it should be further conceptualized what role may clinical notes play in raising the alerts as our current approach only considered structured EHR data. Finally, there are concerns about the possibilities of messaging the provider frequently to the extent where the patient is ignored, which may hurt the patient-provider relationship. In summary, optimizing patient-provider communication in the presence of the *Alerts* and *Collections* features will require a very careful design in the future.

We need to reiterate one more time that the *Collections* and especially the *Alerts* should be approached and designed from the perspective that they are merely tools for supporting sensemaking and decision-making, primarily for the patient but also for the physician. As such, they should never be considered a higher authority than a human expert for taking concrete medical actions. However, they do have the capability to provide context, evidence, and reminders, all very valuable information that could be lacking and easily missed or overlooked by physicians. With this, the *Collections* and *Alerts* should play an important role in health awareness, proactivity, reflection, planning, and advocacy for the patients and serve as context, evidence, and insight enablers for physicians.

Finally, our study was a first step toward identifying patients’ initial reactions to the *Alerts* and *Collections* features. We were mostly focused on evoking patients’ needs, exploring use case scenarios for these features, and surfacing major preferences and concerns related to their use. At this stage, we were not interested in matching patient preferences to patient profiles, so we did not obtain extensive characteristics of the study participants. However, in pursuing refined designs set forth by the directions from this study, it will be important to thoroughly consider the patients’ cognitive, knowledge, or emotional characteristics and find out how these affect the perceptions and attitudes toward the *Alerts* and *Collections*.

### Design Implications

#### Overview

Before we proceed with the design implications, we will list 2 important changes in the modeling and naming of the *Alerts* and *Collections*. First, we can observe that the alerts and collections are, in essence, just groupings of records (ie, data frames that support sensemaking). Both are wrapped with system-generated data or PGD with very similar purposes, which allows us to treat them the same way at the core. Second, the participants associated the alerts with negative meaning but stated that the pattern detection should present the health status of the patient with both negative and positive aspects. For these reasons, we will rename *Alerts* to *Reports*, which carries a more inclusive and neutral tone.

With these adjustments, we offer design implications for (1) contextualizing the reports more deeply for increased actionability and automatically generating the collections for more expedite and exhaustive organization of the EHR data; (2) enabling PGD input in various formats to support more granular organization, richer pattern detection, and learning from experience; and (3) using the reports and the collections for efficient, reliable, and common-ground patient-provider communication.

#### Improvements for Reports and Collections

##### Interpretation of the Reports

There should be 2 main dimensions to the report: what the health status is right now and where it can go. These assessments should be put in a broader context and explain how objectively bad or good things really are with respect to a baseline. In addition, patients should be offered a sense of how hard or easy it would be to maintain the status quo, reach a deterioration point, or improve. These additional, high-level contextualizations of the reports are important for preventing unnecessary panic and providing sometimes much needed relief, motivation, and encouragement.

##### Templates for Collections

To provide automation in determining which records to include in the collections, we propose the idea of collection templates—a mapping between different conditions and records. In the first case, the system would parse the EHR data, determine all possible collections, and prepopulate them with the relevant records. In the second case, the system would allow the user to specify a title for the collection and other metadata and, based on that input, make a best guess at what should be included in the collection. In both cases, the patient should be allowed to modify the collection template to their best interests and knowledge. To support the decision-making of what should stay in the collection or be removed, the system can assign belonging confidence measures to each of the records in the collection. These measurements can also allow the patient to manipulate the precision and recall when a collection template is created.

#### PGD for Data Organization and Logging of Events

##### Granular Data Organization

We should enable features for taxonomizing, deeper nesting of, and linking between reports and collections. For example, deeper nesting of the reports and collections could allow patients to quickly get to more specific topics. In addition, it could help in prioritizing the reports, allowing the patient to focus their attention more narrowly. Furthermore, by enabling linking, we can interrelate individual collections and reuse collections of records throughout different collections. These capabilities are particularly important for diseases that share a common genesis, similar properties, symptoms, and observations. Finally, collections can extend links to reports as a starting point for building more context and collecting additional evidence.

##### Annotations for Issue Resolution Progress

We could allow patients to tag the reports and collections with progress labels in addition to the descriptive labels reported in the results explicitly: topic, urgency, currency, and sentiment. The progress labels should come from a basic taxonomy that describes where the process is in the journey toward its resolution. This small number of labels can then be visually encoded to allow patients to quickly assess progress toward addressing their issues collectively or in isolation and make sense of their priorities.

##### PGD Records

Participants wanted more structure in the data they provided and for those contributions to be treated equally to the data that come from the EHRs. For these reasons, we can dedicate special types of PGD records to life events, manually entered observations and measurements, feelings, the ability to complete tasks in daily life, or data points from devices.

##### Leveraging PGD

This modeling of the data is expected to have secondary benefits. First, with the introduction of PGD records, we are enriching the EHR data and, therefore, enabling potentially more impactful pattern detection. Second, we allow patients to track their daily lives in a structured and searchable format, which can also provide quickly accessible and extremely valuable context and evidence in clinical visits. Finally, within a timeline-based historical view of reports and collections, patients can see if some issues were repeating, when, and how much. By relying on the notes and issue resolution progress annotations, patients can compile and refine strategies for how to address them in the future. In contrast, we could show the evolution of a specific collection or the variety of reports on a particular topic over time. One could imagine how different symptoms, measurements, treatments, and outcomes can vary over time and how the patient has been feeling, coping, and managing the disease in response to that, all captured in the EHR data and PGD. Consequently, this feature could be a tremendously valuable portfolio to learn from previous experiences.

#### Patient-Provider Communication

Similar to previous work [10,14,60], this study showed that patients perceive the provider as a partner in their sensemaking. Future designs of patient-facing sensemaking tools should account for this partnership and provide features that enable the establishment of shared mental models and artifact-based communication.

##### Context and Evidence-Based Communication

For example, one can imagine how a report or collection can be attached to a message and have that message directly reference particular records, notes, or annotations from them. This will provide more granular contextualization for different points in the message and yet keep the full context in the report or collection if needed.

##### Establishing Shared Mental Models

In addition, the tagging of the reports and collections for detailed description and issue resolution progress could be enabled for the patient’s physician as well. With this 2-sided labeling mechanism in place, we could encourage the detection of potential discrepancies in the perceptions of whether certain issues have been addressed or are still open and what the progress is. These could then be further transformed into high-priority talking points via messaging or in clinical visits. Analogously, the collections can be collaboratively edited as well. The physician could also have the right to initiate and populate a collection or suggest adding or removing records for an existing collection, whether created by the system (collection template) or the patient.

##### Message and Task Distribution Among Care Team Members

To avoid physician burnout as a consequence of overwhelming messages, a triaging method should be put in place. For example, clear guidelines should be provided to the patient regarding which care team member should be targeted based on the content of their message. In addition, and because even well-defined guidelines can be difficult to follow, a designated care team member (other than the physician) can perform the triaging manually. Finally, each care team member should have different editing and validation privileges for the patients’ collections and reports. To set common grounds, all messaging, editing, and validating activities should be made available to the entire care team. For transparency of care, this history of activities should also be visible to the patient. By all means, special attention in the design should be paid to whether full transparency applies for all types of activities or whether some should be best left undisclosed to avoid unnecessary overhead in communication, confusion, misguidance, or worry.

### Limitations

This study has several limitations. First, the participant sample did not include older adults, who might have different needs, perceptions, and preferences. Second, we might have introduced certain biases in the mock-ups, although the timing of their introduction and presentation was carefully tailored to avoid this. Third, a lot of the opinions of the participants were based on projecting their expectations for something they had not yet experienced in real life, such as making sense of their own EHR data from multiple providers in a single application, building a collection of records and using it in clinical visits, or making decisions based on a panel of alerts. Fourth, the insights from the participants were obtained based on mock-ups rather than on a fully functional system based on their own EHR and personally generated data, which may have skewed their perceptions or depleted the richness of their feedback. Finally, we did not obtain detailed patient characteristics to map patient profiles to specific needs and preferences.

However, we believe that we came fairly close to our goal of painting a broad picture of what patients’ needs are and how we can design features that support automatic pattern detection and EHR data organization for improved sensemaking and use in real-life scenarios.

### Conclusions

There are untapped opportunities to support automatic pattern detection and organization of EHR data from multiple providers for patient-facing sensemaking applications. In this paper, we investigated the needs patients have with respect to these capabilities and the features that they would prefer for addressing those needs. We learned that patients are open to carefully designed automatic pattern detection with safe and actionable recommendations, which produces a well-tailored and scoped landscape of reports for both potential threats and positive progress. We also learned that patients are willing to contribute their own data in the form of notes, tags, and structured formats to enrich the meaning and enable easier sensemaking of their EHR data through reports and collections. Finally, the study showed that patients wanted to use these artifacts for raising awareness, reflection, and planning but, above all, for contextualized and evidence-based patient-provider communication via messaging or in clinical visits. These findings resulted in design implications for contextualizing more deeply the reports for increased actionability and automatically generating the collections for more expedited and exhaustive organization of the EHR data; enabling PGD input in various formats to support more granular organization, richer pattern detection, and learning from experience; and using the reports and collections for efficient, reliable, and common-ground patient-provider communication.

Although our study was nested in Discovery, the results and design implications can be easily generalized to other existing and future systems. The most important takeaway from this study is that patients need to have a flexible and rich way to organize and annotate their EHR data; be introduced to insights from their data—both positive and negative; and share these artifacts with their physicians during clinical visits or via messaging for establishing shared mental models for goals, priorities, and actions.

Although at this point, we have a better grasp of the direction for supporting automated sensemaking and organization of EHR data for patients, we must not forget that it will take a significant effort until we have a fully functional system. We believe that collaborative efforts and strategizing will benefit the implementation of the insights from our study. With respect to the *Reports* feature, it will take engagement from a wider community to assess the quality requirements for the EHR data for various individual reports, the feasibility and fairness of the data pattern detection algorithms, and the meaningfulness and understandability of the recommendations based on these patterns. Multiple research groups can try to implement different reports (eg, cardiovascular, respiratory, and renal) that can arise from widely adopted clinical guidelines or other trusted sources of knowledge. Furthermore, a gradual approach that primarily targets prevalent conditions and feasible reports should be the starting point so that we can engage wider audiences in realistic and robust evaluations to produce a broader impact. Similarly, any automatic collections should take a similar approach to the reports. For example, a research group can focus on determining which records should be included in a collection for high blood pressure, for kidney failure, and so on. These collaborative efforts can produce a pool of reports and collections that different research groups can borrow from in the implementation of their sensemaking support tools. This will potentially enable faster design-implementation-evaluation cycles and, consequently, the advancement in our knowledge about patients’ use of the reports and collections individually or in collaboration with their physicians.

Although research groups can exert tremendous efforts following the previous guidelines, a key to the success of patient-facing sensemaking tools is the involvement of clinical professionals. In fact, our study pointed out that these tools should, in principle, be regarded as collaboration platforms that improve communication and promote partnership between the patient and physician. With that said, research groups should nurture great relationships with physicians who can contribute valuable insights for the design of such tools. Moreover, to produce usable designs, we will need to engage in evaluations that take place in a clinical setting. For this, physician collaborators will be essential in securing a welcoming setup in their office and even engage as assistants in the research. For successful evaluations, physicians should be willing to sacrifice the comfort of their well-established workflows and have those interrupted by the new sensemaking tools that will inevitably be used at the point of care.

This work is focused on empowering patients by supporting their capabilities to make sense of their EHR data and make these more actionable in real-life scenarios. However, the principles from the Reports could be translated to position the health care team member as the central figure of sensemaking. With this approach, clinicians would be able to obtain reports from an AI agent on the patient’s health status that include their clinical data, demographics, and social determinants of health. In this report, the most likely options for interventions would be suggested based on patient-reported outcomes and clinical outcomes combined with established clinical guidelines. Although this approach does not attempt to shortcut the clinicians as decision makers, it should provide a variety of options to consider as brainstorming for alternatives for individuals in isolation can typically be a cognitively demanding task and result in omitting viable solution paths. In addition, this approach is well aligned with the concept of a learning health care system, which is the guiding star of the latest research endeavors. With recent advancements in AI, we should start preparing for a setting in which there are AI agents that support the work of patients and physicians. In this futuristic setup, which might not be far from now, we can expect that AI agents will help patients in their self-advocacy by assisting them in the sensemaking of their health data and communicating with their providers. In contrast, AI agents will help clinicians decide what is the best care path for the patient and how to communicate that back to them. Having a collaboration between a patient, AI agents, and clinicians will bring an interesting dynamic in the patient-provider communication, which will deserve a deep engagement from researchers. Questions of the type of when and for what tasks AI agents can improve communication, shared decision-making, and the patient-provider relationship will be of high priority.

Finally, although this study produced exciting new design directions for supporting patients’ sensemaking of their EHR data, we have to point out that the features it promotes are disruptive in nature. First, they challenge patients to change the way they interact with their EHR data. Second, they also require adjustments to the existing workflows in the clinical visit and shifting the novice-expert relationship between the patient and the physician toward partnership. We would like to stress that these changes might face amplified resistance and an extended time to take place if we are not extremely careful with our designs and respectful of existing practices. A gradual approach that involves the patients and physicians at every step of the design iteration should be taken. Carefully listening to both stakeholders to deeply understand their needs should help in finding design compromises that will benefit both parties as a team and, ultimately, contribute to a better patient experience and clinical outcomes.

## Abbreviations

AI: artificial intelligence
EHR: electronic health record
FHIR: Fast Healthcare Interoperability Resources
PGD: patient-generated data
RQ: research question
